# Potential impact of vaccination against *Neisseria meningitidis* on *Neisseria gonorrhoeae* in the United States: Results from a decision-analysis model

**DOI:** 10.4161/hv.36221

**Published:** 2014-11-01

**Authors:** Stéphane A Régnier, Jasper Huels

**Affiliations:** 1 Novartis Vaccines & Diagnostics AG; Basel, Switzerland

**Keywords:** decision-analysis model, *Neisseria meningitidis* serogroup B, *Neisseria gonorrhoeae*, vaccination, United States

## Abstract

Components in 4CMenB vaccine against *Neisseria meningitidis* serogroup B have shown to potentially cross-react with *Neisseria gonorrhoeae*. We modeled the theoretical impact of a US 4CMenB vaccination program on gonorrhea outcomes. A decision-analysis model was populated using published healthcare utilization and cost data. A two-dose adolescent vaccination campaign was assumed, with protective immunity starting at age 15 years and a base-case efficacy against gonorrhea of 20%. The 20%-efficacy level is an assumption since no clinical data have yet quantified the efficacy of 4CMenB against *Neisseria gonorrhoea*. Key outcome measures were reductions in gonorrhea and HIV infections, reduction in quality-adjusted life-years (QALYs) lost, and the economically justifiable price assuming a willingness-to-pay threshold of $75,000 per QALY gained. Adolescent vaccination with 4CMenB would prevent 83,167 (95% credible interval [CrI], 44,600–134,600) gonorrhea infections and decrease the number of HIV infections by 55 (95% CrI, 2–129) per vaccinated birth cohort in the USA. Excluding vaccination costs, direct medical costs for gonorrhea would reduce by $28.7 million (95% CrI, $6.8–$70.0 million), and income and productivity losses would reduce by $40.0 million (95% CrI, $8.2–$91.7 million). Approximately 83% of the reduction in lost productivity is generated by avoiding HIV infections. At a cost of $75,000 per QALY gained, and incremental to the vaccine's effect on meningococcal disease, a price of $26.10 (95% CrI, $9.10–$57.20) per dose, incremental to the price of the meningococcal vaccine, would be justified from the societal perspective. At this price, the net cost per infection averted would be $1,677 (95% CrI, $404–$2,564). Even if the cross-immunity of 4CMenB vaccine and gonorrhea is only 20%, the reduction in gonorrhea infections and associated costs would be substantial.

## Abbreviations

4CMenB: Four-component vaccine against *Neisseria meningitidis* meningococcal serogroup B (Bexsero®; Novartis Vaccines and Diagnostics, Siena, Italy)
CDC: US Centers for Disease Control and Prevention
CI: confidence interval
CrI: credible interval (Bayesian analysis)
EVPI: economic value of perfect information
FDA: US Food and Drug Administration
HIV: human immunodeficiency virus
ICER: incremental cost-effectiveness ratio
PID: pelvic inflammatory disease
QALY: quality-adjusted life-year

## Introduction

Gonorrhea is the second most commonly reported sexually transmitted disease in the USA. The Centers for Disease Control and Prevention (CDC) estimates that 820,000 people become infected annually.^1^ The infection is often asymptomatic, particularly in women,^2^ and if untreated the disease can cause a number of adverse outcomes. In women, gonorrhea can lead to pelvic inflammatory disease (PID), infertility, ectopic pregnancy, and chronic pelvic pain; in men, it can cause epididymitis. It can also increase the risk of acquiring human immunodeficiency virus (HIV) infection (infection cofactor per sexual act of 10).^3^ Additional complications include neonatal pneumonia, neonatal conjunctivitis, mortality from ectopic pregnancies, and disseminated gonococcal infections, which can manifest as arthritis-dermatitis syndrome and septic arthritis. The current standard of care is antibiotics (eg intramuscular ceftriaxone plus oral azithromycin and doxycycline),^4^ although there is concern regarding the increasing incidence of antibiotic resistance.

A four-component vaccine (4CMenB; Bexsero®, Novartis Vaccines and Diagnostics, Siena, Italy) against *Neisseria meningitidis* meningococcal serogroup B received Breakthrough Therapy designation from the United States Food and Drug Administration (FDA) in April 2014. It was recently found that the 4CMenB antigens are also present in *Neisseria gonorrhoeae*, suggesting a potential for cross-immunity.^5,6^ Given this potential cross-protection, our objective was to assess the impact of a vaccination campaign with the 4CMenB vaccine on gonorrhea outcomes in the USA.

We developed a decision-analysis model to compare the impact of vaccinating one hypothetical adolescent cohort and the current standard of care for gonorrhea (antibiotics).

## Results

### Health and cost effect

The model predicts that without vaccination, the theoretical adolescent cohort would experience 844,000 gonorrhea infections (95% credible interval [CrI], 439,200–1,399,000) over a lifetime: 500,000 infections in men and 344,000 in women. These infections would be contracted by 622,000 unique individuals, 74% as first infections and 26% as reinfections. Without vaccination, gonorrhea infections would increase the number of HIV cases by 557 (based on the increased risk of HIV infections for gonorrhea-infected individuals). The discounted medical cost was estimated to be $268 million per cohort; the main medical cost driver being incremental costs due to HIV ($138 million), PID ($74 million, including sequelae), and treatment ($56 million). Income lost due to gonorrhea was estimated to be $75 million per cohort. Additionally, income of $301 million was predicted to be lost due to incremental HIV cases. A total of 14,106 undiscounted quality-adjusted life-years (QALYs) were predicted to be lost per cohort, driven by QALYs lost because of gonorrhea-related infertility (5,933 or 42% of lost utilities), incremental HIV infection (3,873 or 27% of lost utilities), and chronic pelvic pain (3,383 or 24% of lost utilities).

Assuming a vaccine efficacy of 20% against gonorrhea and an average duration of effect of 10 years, the model predicts that the vaccination of one theoretical birth cohort could prevent 83,167 (95% CrI, 44,600–134,600) gonorrhea infections over a lifetime (70,849 if discounted). The vaccination program could also save 1,265 (95% CrI, 407–3,169) QALYs per vaccinated cohort (1,052 if discounted). The direct medical costs were modeled to decrease by $28.7 million (discounted) (95% CrI, $6.8–$68.0 million), and income and productivity losses by $40.0 million (95% CrI, $8.2–$91.7 million).

From the societal perspective, without considering the vaccine's effect on meningococcal disease, the economically justifiable price per dose for an effect on gonorrhea was $26.10 (95% CrI, $9.10–$57.20), which translates into incremental (to meningococcal program) vaccination costs of $148 million. Accounting for the reduction in direct medical costs, the incremental net cost of vaccination was $119 million per vaccinated cohort. Therefore, the net cost per gonorrhea infection averted would be $1,677 (95% CrI, $404–$2,564). The exclusion of income lost as a result of gonorrhea sequelae only decreased the economically justifiable price by $1.20 per dose.

### Sensitivity analyses

**Figure 1.**
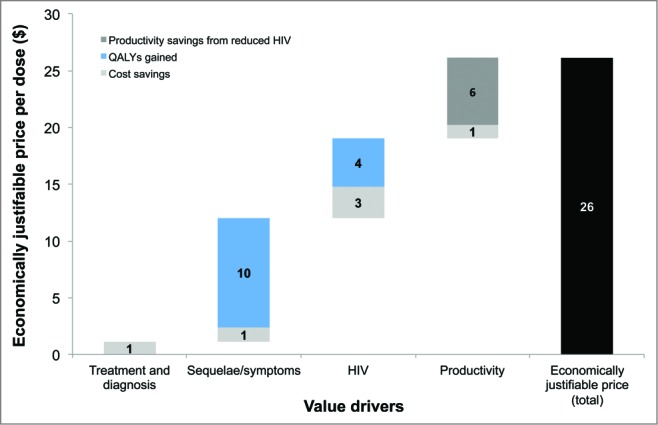
Drivers of the economic value of the vaccine. Total values are shown from healthcare provider and societal perspectives. HIV: human immunodeficiency virus; QALY: quality-adjusted life-year.

**Figure 2.**
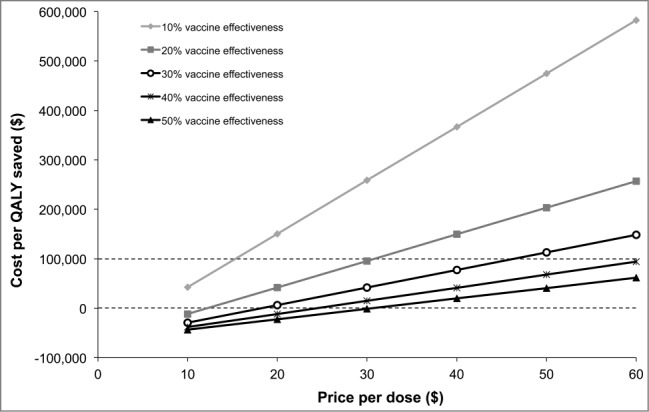
Impact of vaccine effectiveness and price per dose on the cost-effectiveness of a gonorrhea indication. QALY: quality-adjusted life-year.

A substantial proportion of the costs avoided and QALYs gained would be driven by the reduction in HIV infections (**Fig 1**). If it is assumed that gonorrhea does not increase the risk of contracting HIV, the economically justifiable price would only be $13.20/dose. If the disutility associated with infertility and chronic pelvic pain was assumed to last for 20 years instead of 10 years, the economically justifiable price would be $32.70 per dose. **Figure 2** shows the impact on cost-effectiveness of modifying vaccine effectiveness and price. The incremental cost-effectiveness ratio (ICER) improves substantially if the effectiveness increases from 10% to 20%. At a price per dose of $10 ($20 per course), the vaccine would be cost saving if the efficacy is 20%. There was a diminishing return in terms of ICER improvements when effectiveness increased to higher levels. For all prices tested, the differences were relatively small between efficacies of 50% and 40%.

**Figure 3.**
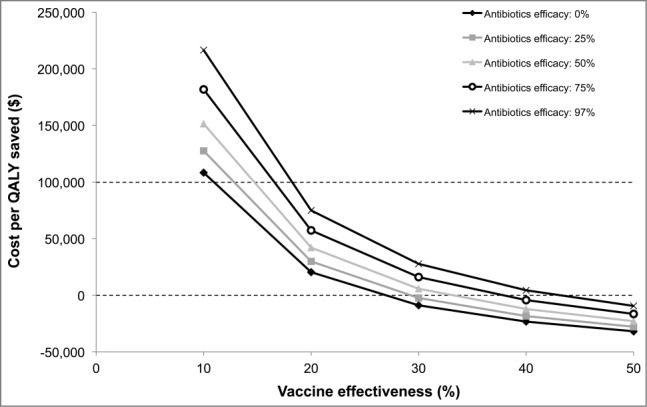
Impact of antibiotic efficacy and vaccine effectiveness on the cost-effectiveness of a gonorrhea indication. A price of $26.10 per dose is assumed. QALY: quality-adjusted life-year.

Antibiotic efficacy also has a substantial impact on the vaccine's cost-effectiveness, especially if the effectiveness of the vaccine is low (**Fig 3**). Also, if the cost of new antibiotic treatment (for the same efficacy) was twice as high as today's, the economically justifiable price of the vaccine would be $27 per dose; similarly, if the cost of new antibiotic treatment was 5 or 10 times as high as today's, the economically justifiable price of the vaccine would be $31 and $36 per dose, respectively.

If the incidence data were based on reported cases without estimates for underreporting (ie 299,000 gonorrhea cases per cohort instead of 844,000 in the base case), the number of infections prevented would be 30,417 and the economically justifiable price would be $10.50 per dose.

**Figure 4.**
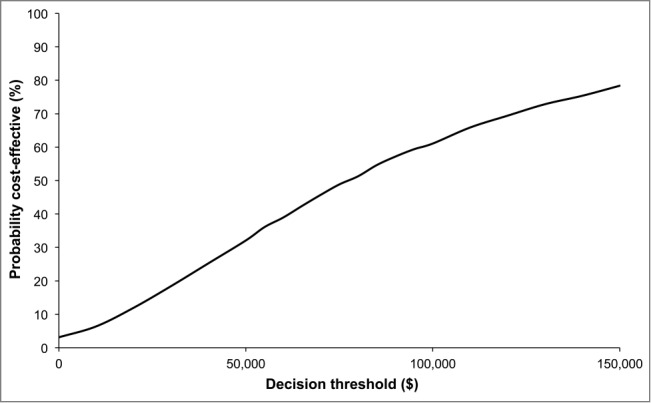
Cost-effectiveness acceptability curve (assuming a price per dose of $26).

The cost per QALY gained was below $100,000 for 61% of the simulations and below $50,000 in 32% (**Fig 4**); 3% of simulations were deemed to be cost saving. A decision-maker will be willing to pay for the benefits if the probability of the decision being cost-effective is high. Conversely, if the probability is low, the decision-maker will not be willing to pay. For each cost per QALY threshold, there will be a probability that the decision-maker will make the wrong decision (eg not recommend a cost-effective treatment). The cost of such a decision is the economic value of perfect information (EVPI). In our model, it is $24.0 million if the willingness-to-pay is $75,000 per QALY gained (Supplemental Digital Content 1).

## Discussion

The incremental price of $26.10 per dose only applies to vaccines, such as 4CMenB, that have shown to potentially cross-react with *Neisseria gonorrhoea*. For instance, it may not apply to vaccines against meningitis serogroups ACWY.

The cost of diagnosis and antibiotic treatment of gonorrhea is low. Despite the high efficacy (97%) and low price of antibiotics in the base case, there is value in using a preventive rather than a curative approach to gonorrhea. This is mainly due to the low treatment rate (see Methods section) which is mainly driven by the high percentage of patients (especially women) who are asymptomatic. Consequently, the value of a vaccination against gonorrhea is mainly in preventing the long-term consequences of the disease for women (ie chronic pelvic disease and infertility), and in decreasing the number of HIV infections that are facilitated by gonorrhea infection. The productivity loss that results from the complications of gonorrhea is also small; however, productivity lost due to HIV infection is substantial. This is driven by the high loss of income for people with HIV (∼$1 million/individual).

In our model, the base case predicts a higher number of HIV cases associated with gonorrhea than calculated by Chesson and Pinkerton^3^ (557 vs. 430). This is because our model assumes a more recent and higher incidence of gonorrhea infections (844,000 vs. 650,000). The link between gonorrhea and HIV infections is expected to continue to exist because the HIV prevalence has been increasing despite the decreasing transmission rate of HIV.

If the resistance of gonorrhea to antibiotics is high, a vaccine with an efficacy against gonorrhea of just 10% could be cost-effective at a price of $26 per dose. The rationale behind this is that the burden of disease will be much higher if antibiotics have a limited impact, and a 10% reduction in the burden of disease could have a considerable impact. The differences between the scenarios modeling low and high antibiotic resistance are somewhat limited (**Fig 3**). There are 2 main reasons for this. Firstly, half of infections are currently not treated successfully so antibiotic resistance does not have an impact on these. Secondly, the model is static with a fixed incidence.

The cost-effectiveness acceptability curve is relatively flat, which reflects the fact that there are many uncertainties for key input parameters. More specifically, a uniform distribution with a large range of potential values was used for the QALYs lost per HIV case and the cost of a case of HIV. As a consequence, the EVPI per vaccinated cohort is substantial (up to $24.1 million). The value of conducting research to reduce the uncertainties depends on the decision-maker's timeframe. For instance, if the timeframe is 10 years, it would make economic sense to spend up to $194 million on research to eliminate the uncertainties if the willingness-to-pay is $75,000 per QALY gained.

The gonorrhea incidence rates vary dramatically, not only by age but also by ethnic group and geography. For instance, the rate of reported disease among non-Hispanic blacks is 15 times higher than among non-Hispanic whites. Therefore, the cost-effectiveness of the vaccination campaign will also vary substantially by ethnic group and geography.

Since the primary reason for 4CMenB vaccination is the prevention of meningococcal disease, the level of the impact on gonorrhea does not depend on the awareness of gonorrhea disease and its consequences. Instead, it will be based on the awareness of meningococcal disease (see Methods for vaccination rate assumptions). Therefore, a low awareness of gonorrhea disease and its consequences will not prevent the medical benefits of the vaccine from being achieved.

This analysis took a United States societal perspective. However, the method could be applied in other countries. The most impactful assumptions to be updated are gonorrhea incidence, the cost of HIV treatment and the lifetime productivity losses linked to HIV.

### Limitations

The main limitation of the model is that the efficacy of the vaccine is not known (we made the assumption that the efficacy of the vaccine against gonorrhea was likely to be much lower than its efficacy against serogroup B meningococcal disease: 20% vs 78%). Sensitivity analyses were presented to assess the efficacy's impact on cost-effectiveness. Additional research should be conducted to estimate the real-life effectiveness. A number of key inputs (eg QALYs lost due to infertility) came from expert-panel estimates^7^; estimates derived from the general population would be more appropriate. In our model, the additional infections averted by preventing one case (ie the herd effect of interrupting disease transmission) were taken into account using a simplistic factor of 0.5 for all cases averted; as soon as clinical evidence is available that allows evidence-based dynamic modeling of the indirect effect, the cost-effectiveness of such an approach will warrant additional study.

The vaccination rate was assumed to be the same for men and women, and for at-risk groups (eg men who have sex with men) and those not at risk. The rationale was that at-risk sexual behavior would not have developed by the time of vaccination.

## Conclusions

Even with a low effectiveness (eg 20%) against gonorrhea, a US vaccination program against the serogroup B meningococcal disease using 4CMenB would substantially reduce the number of gonorrhea infections, and would justify an incremental price per dose of $26.10 ($52.20 per course), without considering the vaccine's effect on meningococcal disease. However, because of uncertainties in input parameters used in the model, the credible interval is wide; additional research should be conducted to narrow this. More specifically, the vaccine's efficacy against gonorrhea, and uncertainties around the number of incremental HIV infections due to gonorrhea, the number of QALYs lost due to HIV, and the costs of HIV should be addressed. Even if the uncertainties were resolved, it is unclear that the Advisory Committee on Immunization Practices (ACIP) would consider a benefit as low as 20% in its economic evaluation. The timing of the evidence generation may also matters. If the clinical data quantifying the 4CMenB's efficacy against gonorrhea comes after the meningococcal evaluation, it is unclear that the manufacturer will be able to subsequently raise the vaccine's price.

## Methods

### Model

The economically justifiable price from the societal perspective was calculated using the formula:

$$pd+Ad=\frac{\lambda *Qg+Ms+Ps}{Co*Ra*Ds}$$

Where *Pd* is the economically justifiable price per dose, *Ad* the administration fees per course, λ the willingness-to-pay for 1 QALY gained, *Qg* the number of QALYs gained from vaccination, *Ms* the medical cost savings derived from vaccination, *Ps* the productivity cost savings derived from vaccination, *Co* the cohort size, *Ra* the vaccination rate, and *Ds* the number of doses required per vaccinated individual. Adolescents were assumed to receive 2 doses per course.^8^ No wastage, advertising, or adverse-event costs were assumed. As the objective of our model was to evaluate the incremental impact on gonorrhea of a meningococcal vaccination, *Ad* was zero. The economically justifiable price was additionally calculated taking into account income lost, but results are also presented without any impact on productivity.

There is no official λ in the USA. However, an intervention is usually regarded as cost-effective if the cost per QALY gained is below $50,000 and not cost-effective if it exceeds $100,000.^9^ In the base case, a willingness-to-pay of $75,000 per QALY gained was assumed.

**Figure 5.**
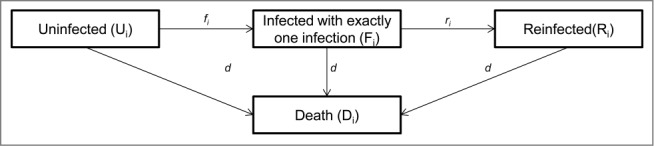
Markov model simulating the individuals without infection, with one infection, and multiple infections per cycle i.

We used a Markov chain to simulate gonorrhea infections and reinfections (**Fig 5**). This structure avoids double-counting benefits by distinguishing the costs that are linked to infections (including reinfections) from those associated with sequelae developing in infected women (regardless of the number of reinfections). Each Markov cycle lasted for 1 year and the time horizon was lifetime. Two models were developed: one for men and one for women. The size of the vaccinated adolescent cohort of age 15 years (2,047,000 men and 1,957,000 women) was based on 2011 United States Census Bureau data.^10^

The model assumed that a routine vaccination against meningococcus (including serogroup B) would be implemented in the physician office setting. Currently, 4CMenB is approved in over 30 countries but not in the USA. In our model, we assumed that 4CMenB or the next generation of 4CMenB will be approved in the future in the USA. The model considered is a static model that includes the benefits to the individuals who are vaccinated, in combination with estimates for the population-level benefits that – similar to other sexually transmitted diseases – result from the decrease in prevalence due to vaccination.^11^ An adolescent vaccination rate of 70.5% was assumed, similar to the 2011 vaccination rate against meningitis serogroup ACWY.^12^

The analyses were conducted using the WinBUGS statistical program (version 1.4; Medical Research Council Biostatistics Unit, Cambridge, UK).

### Medical assumptions

#### Infection rate

In the absence of published estimates for each of the cycles, we used the reinfection rate estimated by Gunn and colleagues^13^ in the first year. A reinfection rate *r* of 4.5% per cycle was assumed at the peak of disease incidence (age 18–24 years). For other age groups, the reinfection rate was adjusted in proportion to the incidence (ie the reinfection rate depends on the Markov cycle *i*). Recent estimates^1^ of disease incidence at age 15–39 years (ie the sum of annual infections and reinfections) were used. The breakdown of the incidence of gonorrhea between ages 15 and 39 years (**Table 1**) was calculated using the percentage of reported cases per age group in 2011, collated by the CDC.^14^ The incidence of gonorrhea in each age group between ages 40 and 65 years was calculated assuming that the level of under-reporting was comparable to that at age 15–39 years. Incidences for ages above 65 years were not included in the model because they had a minimal impact on the results (low incidence and low compounded discounted values). Death rates were assumed to be the same for gonorrhea and non-gonorrhea health states, and were varied by Markov cycle according to National Vital Statistics Reports data.^15^

**Table 1.** Incidence of gonorrhea infection per 100,000 for hypothetical cohort (including under-reporting)

Age group (years)	Men	Women
15–19	933	1,278
20–24	1,611	1,273
25–29	922	518
30–34	565	238
35–39	332	113
40–44	243	58
45–54	134	22
55–64	47	4

Sources: References 1,14,15.

#### Treatment rate

**Figure 6.**
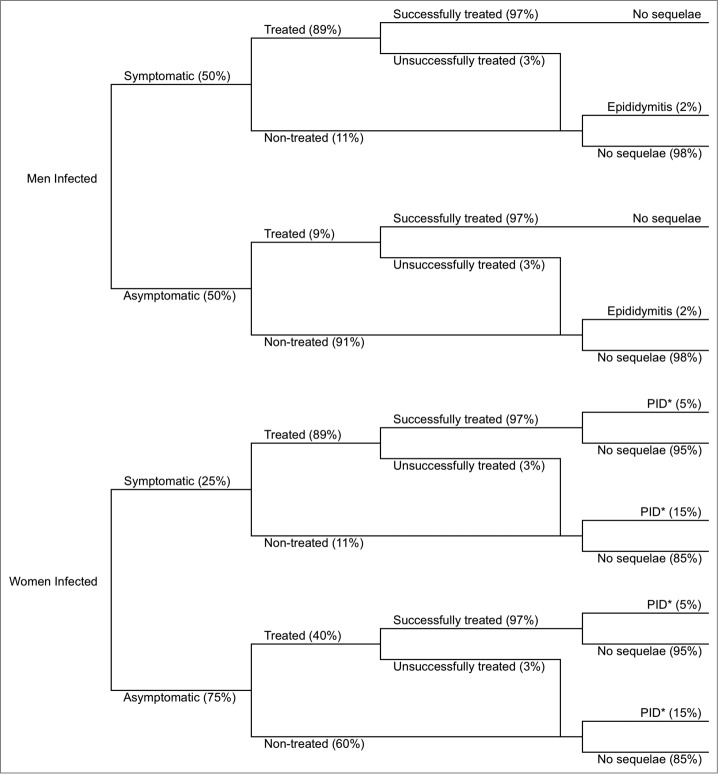
Disease pathway. PID: pelvic inflammatory disease. *PID can lead to infertility, chronic pelvic pain, and infertility. Sources: References 2,17,19,20.

**Figure 6** shows the gonorrhea treatment pattern for men and women: 50% of infections in men and 25% of infections in women are symptomatic^2^; the treatment rate of symptomatic cases was assumed to be 89% for both sexes.^2^ Asymptomatic men are only treated in 9% of cases; asymptomatic women are treated in 40% of cases.^2^ Hereon, ‘untreated’ individuals refers not only to individuals whose treatment was not efficacious but also to those who did not receive treatment.

#### Efficacy and safety

There are no data on the level of cross-protection between *N meningitidis* and *N gonorrhoeae*, so the base case assumed that vaccination would reduce the probability of gonorrhea-associated events by only 20% starting at age 15 years. This assumption is arbitrary but its rationale was that the vaccine efficacy with regard to gonorrhea was likely to be much lower than that of the vaccine against meningococcal serogroup B disease, where 78% (95% CI, 63%–90%) of *N meningitidis* serogroup B strains are potentially susceptible to vaccine-induced antibodies.^8^ Since the base case efficacy assumption is theoretical, the efficacy level was varied in sensitivity analyses. The average duration of protection of 10 years was extrapolated from clinical study data^16^ (in which at least 96% of participants had serum bactericidal antibody titers of 4 or more, 6 months after receiving the first of their 2 doses of 4CMenB). We assumed that each case of gonorrhea prevented through the vaccination program prevented an additional 0.5 cases in the general population.^11^ The model assumed that routine vaccination against meningococcus B was implemented so only incremental benefits on gonorrhea were assumed (no incremental side effects were assumed). The efficacy of antibiotics in treating gonorrhea infections was assumed to be 97% in the base case.^17^

#### Disease consequences

**Table 2** summarizes key assumptions for healthcare utilization associated with *N. gonorrhoeae* infection. For women the main complication is PID.^2^^,^^17-20^ Depending on whether or not they are successfully treated, the base case assumed that between 5% and 15% of infected women developed such complications.^19^ PID's sequelae in women were also considered.^17,21^

**Table 2.** Clinical data for infection with *N. gonorrhoeae*

Parameter	Mean estimate	Range*	Distribution	Source
Antibiotic efficacy	97%	95–100%	Uniform	17
Additional protection from one prevention	0.5 cases	-	Fixed	11
Women				
Incidence	0.69% of women aged 15–39 years	0.32–1.21%	Beta	1
Symptomatic	25% of infected women	-	Fixed	2
Treated	89% of symptomatic	-	Fixed	2
	40% of asymptomatic	-	Fixed	2
PID	5% of treated	0–10%	Uniform	19,20
PID	15% of untreated	5–30%	Uniform	19,20
Ectopic pregnancy	7.5% of women with PID	2–10%	Uniform	17,21
Chronic pelvic pain	18% of women with PID	15–20%	Uniform	17,21
Infertility	20.5% of women with PID	11–30%	Uniform	17,21
Men				
Incidence	0.89% men aged 15–39 years	0.30–1.79%	Beta	1
Symptomatic	50% of infected	-	Fixed	2
Treated	89% of symptomatic	-	Fixed	2
	9% of asymptomatic	-	Fixed	2
Urethritis	84% of symptomatic	-	Fixed	7
Epididymitis	2% of untreated	1–4%	Uniform	20
Incremental HIV infection	0.00066 per gonorrhea infection	0–0.00132	Uniform^†^	3
HIV-positive individuals with viral suppression	25% of HIV infected	-	-	32
QALYs lost				
PID	0.00877 per episode	-	Fixed	7
Ectopic pregnancy	0.02973 per pregnancy	-	Fixed	7
Chronic pelvic pain	0.083 per year	0.044–0.122	Uniform	22
Infertility	0.1656 per year	0–0.3312	Uniform^††^	7
HIV	6.95 (lifetime)	4.85–9.05	Uniform	26
Epididymitis	0.00920 per episode	-	Fixed	7
Urethritis	0.00285 per episode	-	Fixed	7
Disutility duration				
Chronic pelvic pain	10 years	-	Fixed	17
Infertility	10 years	-	Fixed	17

HIV: human immunodeficiency virus; PID: pelvic inflammatory disease; QALY: quality-adjusted life-year.

*Min–max for uniform distribution; 95% confidence interval for β distribution.

^†^Confidence interval centered around the base case value.

^††^The authors assumed a uniform distribution centered around the reported mean.

In men, the vast majority of symptomatic cases (84%) involve urethritis.^7^ Approximately 2% of untreated men develop epididymitis.^20^ Successfully treated men were assumed not to develop epididymitis.^20^

#### Impact on quality of life

Disutilities for symptoms and sequelae used in the model are summarized in Supplemental Digital Content 2. The disutility from chronic pelvic pain was calculated from a survey of 17,927 households.^22^ Disutilities due to PID and infertility were calculated using the input of experts from the Institute of Medicine^7^ (see Supplemental Digital Content 2 for detailed calculations), who estimated the health utility index of infertility due to gonorrhea to be 0.82. This estimate was used because of the lack of empirical data for the general population. The estimates were adjusted with population sex- and age-adjusted weights.^23^ The average utility value for the population was assumed to be 0.93 for men and 0.92 for women aged 15–24 years.^23^ The disutility associated with infertility was not applied to women who were voluntarily infertile (9%) or who had unwanted pregnancies (14%) as we assumed that no utility would have been lost if those women were to become infertile (conservative assumptions).^24,25^ The duration of the quality-of-life decrement due to chronic pelvic pain and infertility was assumed to be 10 years.^17^

Yeh and colleagues found that chronic pelvic pain, ectopic pregnancy, and infertility develop 2–10 years after initial upper genital tract infection.^21^ In the base case, we assumed that complications from PID developed 5 years after initial infection.

The loss of QALYs for each episode of epididymitis was assumed to be 0.0092.^7^

#### Co-infection

Gonorrhea is a significant cofactor for HIV transmission and may increase the risk of HIV transmission. Chesson and Pinkerton used an infection cofactor per sexual act of 10.^3^ The probability that a case of gonorrhea in the USA will facilitate a new case of HIV was estimated to be 0.00066 (range 0–0.57)^3^; the incremental risk for a treated individual was estimated to be 0.000058.^3^ The incremental risk for an untreated individual was 0.001239 (so that the average transmission risk across successfully and untreated individuals remained at 0.00066 in the base case). The number of QALYs lost over a lifetime per HIV case has been estimated to be 6.95.^26^

### Cost assumptions

Cost estimates (**Table 3**) were derived from published sources and adjusted to 2012 US dollars, based on the medical care component of the Consumer Price Index. Ideally, costs rather than charges should be used to better reflect “the true opportunity cost to society of the resources used”^27^; however, charges are often reported in the literature.

**Table 3.** Cost estimates

Cost per case	Mean Estimate (US$)	Distribution	Sources
Direct medical cost of acute gonorrhea*			
Men	177	Gamma	2,28,29
Women	170	Gamma	2,28,29
Pelvic inflammatory disease (lifetime)	3,420	Gamma	2
Direct medical costs/ HIV infection (lifetime)	325,500 (range: 244,600–405,300)	Uniform	26
Epididymitis	334	Gamma	2
Productivity loss (per individual)			
Untreated men	39		11
Untreated women	195		11
Treated men/women	201		30
Lifetime productivity lost per HIV case	947,309		33

HIV: human immunodeficiency virus.

*Outpatient visits, diagnostics, antibiotic costs.

#### Medical Costs

Approximately 27% of diagnoses and treatments for gonorrhea occur in private healthcare settings, while 73% take place in other settings such as hospitals and family planning clinics.^2^ The average cost of diagnosis and treatment across private and public settings is $177 per treated man and $170 per treated woman.^28,29^

The lifetime medical cost of the consequences of one case of PID, including infertility, ectopic pregnancy, and chronic pelvic pain, was estimated to be $3,420.^2^ The cost of one case of epididymitis in men was estimated to be $334.^2^ The lifetime cost of an incremental HIV case was estimated at $325,500^26^ (this includes individuals who receive antiretroviral therapy and those who do not).

#### Vaccination program

Vaccination costs were calculated by multiplying the cohort size, coverage rate, number of doses per individual, and the vaccine costs.

#### Income loss

An income of $39 (men) and $195 (women) was assumed to be lost for untreated individuals and $201 for treated individuals (both men and women).^2,30^ Compared with the general population, there is no increase in mortality for HIV-positive patients receiving antiretroviral therapy who have a recent undetectable viral load, and who maintain CD4+ cell counts at ≥500 cells/μL or whose CD4+ counts recover to this level.^31^ However, 75% of individuals with HIV do not achieve suppression of viral load^32^; income loss due to higher mortality was only assumed for these individuals. We used the lifetime income loss for an HIV-positive patient estimated by Hutchinson et al.^33^ Income loss reported in the literature was adjusted using the All-items Consumer Price Index.

### Discounting

The present value of future costs and benefits was calculated using a discount rate of 3%.

### Sensitivity analysis

As the effectiveness of the 4CMenB vaccine against *N. gonorrhoeae* is unknown, values for efficacy were varied over a wide range in univariate sensitivity analyses. The impact of increased antibiotic resistance was also assessed in 2 different ways: (i) in one set of scenarios, we assumed that the antibiotics will have a reduced probability of treating the disease successfully; (ii) we also assumed that new drugs will be discovered that will be as effective as those currently on the market, but we assumed that the price of such compounds would be higher than that for today's treatments. A probabilistic sensitivity analysis using a Monte Carlo simulation was conducted using the distribution of key variables (**Table 3**). Results from 3,000 simulations were used to determine CrIs for key outcomes. If no standard errors or ranges were provided for the mean estimate, a standard error equal to the mean was assumed.

## Supplementary Material

Economic_value_of_perfect_information__EVPI__per_cohort.pdf

Calculation_of_QALYs_lost_per_case_or_per_event.docx

WinBUGS_code_used_in_the_analysis.docx
