# Supplementary figures and images for: Potential impact of vaccination against Neisseria meningitidis on Neisseria gonorrhoeae in the United States: Results from a decision-analysis model
Source: Hum Vaccin Immunother. 2014 Nov 1;10(12):3737–45. doi: 10.4161/hv.36221 (PMC4514066; doi:10.4161/hv.36221)

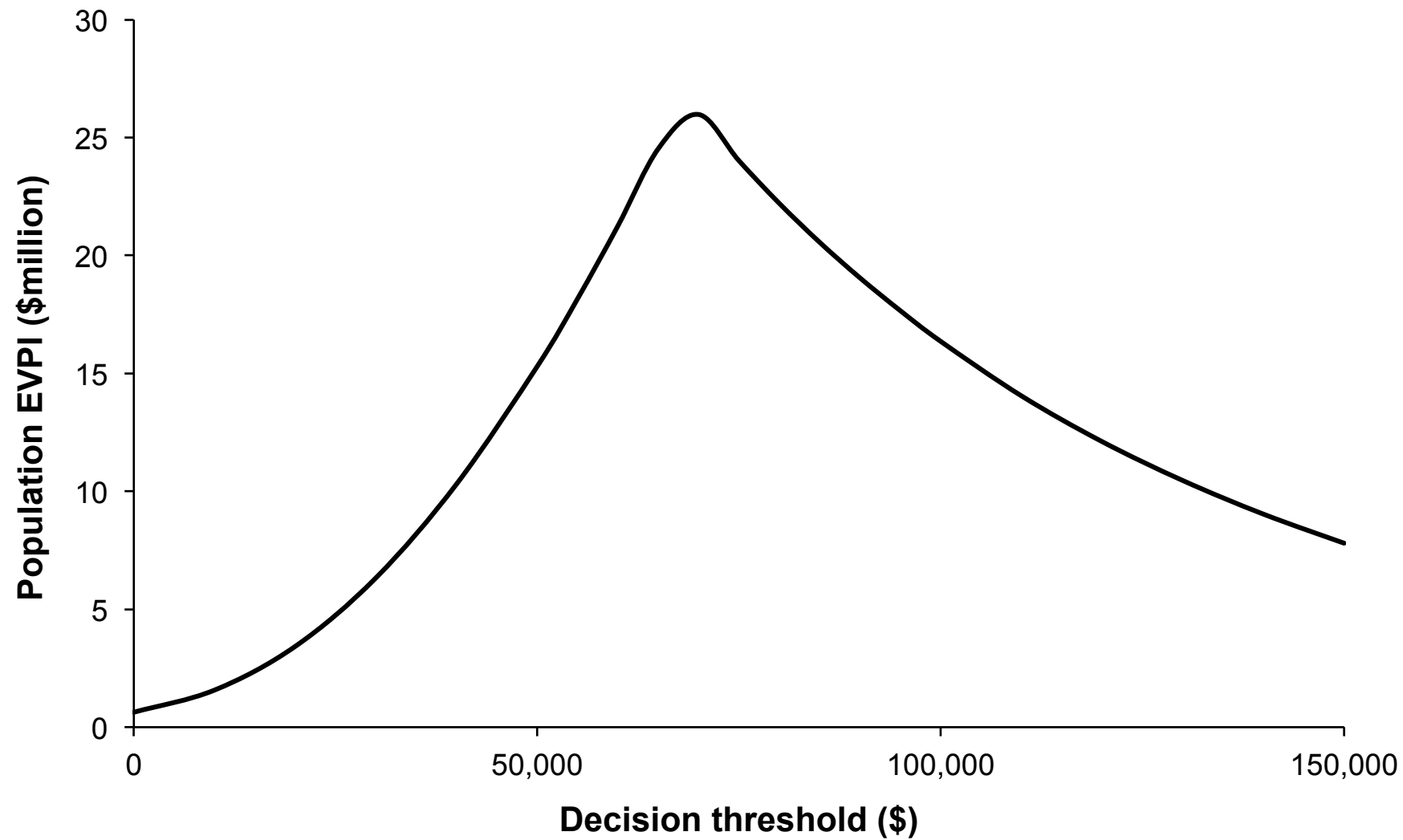

Supplement: Economic_value_of_perfect_information__EVPI__per_cohort.pdf [file khvi-10-12-975067-s001.pdf]
