# Supplementary material for: Potential impact of vaccination against Neisseria meningitidis on Neisseria gonorrhoeae in the United States: Results from a decision-analysis model
Source: Hum Vaccin Immunother. 2014 Nov 1;10(12):3737–45. doi: 10.4161/hv.36221 (PMC4514066; doi:10.4161/hv.36221)
Supplement: Calculation_of_QALYs_lost_per_case_or_per_event.docx [file khvi-10-12-975067-s002.docx]

**Supplemental Digital Content 2: Calculation of QALYs lost per case or per event**

| **Event** | **Disutility (*A*)** | **Age adjustment (*B*)** | **Duration (years) (*C*)** | **QALYs lost per case =** $\sum_{\boldsymbol{i=0}}^{\boldsymbol{i=C-1}} \frac{\boldsymbol{A*B}}{\boldsymbol{(1+r)}^{\boldsymbol{i}}}$ |
| --- | --- | --- | --- | --- |
| Chronic pelvic pain | 0.083 | 1 | 10 | 0.7992 |
| Infertility | 0.18 | 0.92 | 10 | 1.4550 |

| **Event** | **Disutility (*A*)** | **Age adjustment (*B*)** | **Duration (days) (*C*)** | **QALYs lost per episode = A*B*C/365** |
| --- | --- | --- | --- | --- |
| Urethritis | 0.16 | 0.93 | 7 | 0.00285 |

| **Event** | **Disutility (*A*)** | **Age adjustment (*B*)** | **Duration (days) (*C*)** | **QALYs lost per episode (D = A*B*C/365)** | **% (E)** | **Aggregated QALYs lost (weighted average of D with E)** |
| --- | --- | --- | --- | --- | --- | --- |
| **Epididymitis** |  |  |  |  |  | **0.00920** |
| *Outpatient* | 0.54 | 0.93 | 7 | 0.009631 | 90 |  |
| *Inpatient* | 0.3 | 0.93 | 3 | 0.005351 | 10 |  |
| **Ectopic pregnancy** |  |  |  |  |  | **0.02973** |
| *Outpatient only* | 0.42 | 0.92 | 28 | 0.29642 | 50 |  |
| *Inpatient* |  |  |  |  | 50 |  |
| Inpatient first | 0.77 | 0.92 | 3 | 0.005822 |  |  |
| Outpatient after inpatient | 0.34 | 0.92 | 28 | 0.023996 |  |  |
| **Pelvic inflammatory disease** |  |  |  |  |  | **0.00877** |
| *Outpatient only* | 0.37 | 0.92 | 10 | 0.009326 | 50 |  |
| *Inpatient* |  |  |  |  |  |  |
| No-surgery | 0.43 | 0.92 | 4 | 0.004335 | 37.5% |  |
| Surgery | 0.54 | 0.92 | 2 | 0.002722 | 12.5% |  |
| Outpatient after inpatient | 0.17 | 0.92 | 10 | 0.004285 | 50% |  |
