# Supplementary material for: Potential impact of vaccination against Neisseria meningitidis on Neisseria gonorrhoeae in the United States: Results from a decision-analysis model
Source: Hum Vaccin Immunother. 2014 Nov 1;10(12):3737–45. doi: 10.4161/hv.36221 (PMC4514066; doi:10.4161/hv.36221)
Supplement: WinBUGS_code_used_in_the_analysis.docx [file khvi-10-12-975067-s003.docx]

**WinBUGS code [for reviewers only]**

**#MODEL**

model

{

#Probability of getting infected between 15 and 64 years

for(i in 1:ns){

for(k in 1:nbincgrp){

for(l in 1:nbcohortspergroup){

probinfected[i,(k-1)*nbcohortspergroup+l]<-incidence[i]*pctcasesperagegroup[i,k]/(cohortsizeagegroup[i,k])

}

}

#No infections above 65 years of age

for(k in (nbincgrp)*nbcohortspergroup+1:nt){

probinfected[i,k]<-0

}

}

#death rate for each age stage

for(i in 1:ns){

for(k in 1:12){

for(l in 1:nbcohortspergroup){

deathratetime[i,(k-1)*nbcohortspergroup+l]<-deathrate[i,k]

}

}

}

#Reduction in probability of getting infected if vaccinated

for(t in 1:nt){

efficacy[t]<-exp(-(t-1)/efficmeanduration)*efficacyrate

}

#Number of infections per time period with and without vaccination

for(i in 1:ns){

#Calculate population size (i.e. cohort size at age 15 minus deaths)

cohortsize[i,1]<-cohortvac[i]

for(t in 2:nt){

cohortsize[i,t]<-cohortsize[i,t-1]*(1-deathratetime[i,t-1])

}

for(t in 1:nt){

#Without vaccination

#Number of infections, symptomatic infections and asymptomatic infections

infectionsnovac[i,t]<-probinfected[i,t]*cohortsize[i,t]

symptomaticnovac[i,t]<-symptomaticprob[i]*infectionsnovac[i,t]

asymptomaticnovac[i,t]<-(1-symptomaticprob[i])*infectionsnovac[i,t]

#Number of sucessfully treated and unsuccessfully treated (incl. non-treated) infections

treatednovac[i,t]<-(symptomaticnovac[i,t]*treatSprob[i]+asymptomaticnovac[i,t]*treatASprob[i])*successrate

nontreatednovac[i,t]<-symptomaticnovac[i,t]*(1-treatSprob[i])+asymptomaticnovac[i,t]*(1-treatASprob[i])+(symptomaticnovac[i,t]*treatSprob[i]+asymptomaticnovac[i,t]*treatASprob[i])*(1-successrate)

#Number of incremental HIV cases due to gonorrhea

HIVindirectnovac[i,t]<-infectionsnovac[i,t]*HIVprob

#With vaccination

#Number of infections, symptomatic infections and asymptomatic infections

infectionswithvac[i,t]<-infectionsnovac[i,t]*(1-efficacy[t]*vaccinationrate)

#Number of infections prevented without indirect protection

infectionspreventedvaccinated[i,t]<-infectionsnovac[i,t]-infectionswithvac[i,t]

#Number of infections prevented including indirect protection

infectionsprevented[i,t]<-infectionspreventedvaccinated[i,t]*(1+addtransmittedcase)

symptomatic[i,t]<-symptomaticprob[i]*infectionsprevented[i,t]

asymptomatic[i,t]<-(1-symptomaticprob[i])*infectionsprevented[i,t]

#Number of sucessfully treated and unsuccessfully treated (incl. non-treated) infections avoided due to vaccination

treated[i,t]<-(symptomatic[i,t]*treatSprob[i]+asymptomatic[i,t]*treatASprob[i])*successrate

nontreated[i,t]<-symptomatic[i,t]*(1-treatSprob[i])+asymptomatic[i,t]*(1-treatASprob[i])+(symptomatic[i,t]*treatSprob[i]+asymptomatic[i,t]*treatASprob[i])*(1-successrate)

#Number of incremental HIV cases avoided due to vaccination

HIVindirect[i,t]<-infectionsprevented[i,t]*HIVprob

}

}

#Number of uniquely infected people per time period

for(i in 1:ns){

#In the first period, there are only first infections (i.e. number of infections=number of infected)

#twoinfection variables count the number of people who had 2 or more infections

oneinfectionnovacc[i,1]<-infectionsnovac[i,1]

twoinfectionnovacc[i,1]<-0

firstinfectionnovacc[i,1]<-infectionsnovac[i,1]

oneinfectionwithvacc[i,1]<-infectionswithvac[i,1]

twoinfectionwithvacc[i,1]<-0

firstinfectionwithvacc[i,1]<-infectionswithvac[i,1]

dead[i,1]<-0

for(t in 2:nt){

#Without vaccination

#Reinfection rate per period is normalized based on the number of infections per period

rirate[i,t]<-reinfectionrate*probinfected[i,t]/max(probinfected[i,1],probinfected[i,1+nbcohortspergroup])

oneinfectionnovacc[i,t]<-oneinfectionnovacc[i,t-1]*(1-rirate[i,t])+infectionsnovac[i,t]

twoinfectionnovacc[i,t]<-twoinfectionnovacc[i,t-1]+oneinfectionnovacc[i,t-1]*rirate[i,t]

#Number of people who get infected for the first time in perid t

firstinfectionnovacc[i,t]<-oneinfectionnovacc[i,t]-oneinfectionnovacc[i,t-1]

#With vaccination

riratewithvacc[i,t]<-rirate[i,t]*(1-efficacy[t]*vaccinationrate)

oneinfectionwithvacc[i,t]<-oneinfectionwithvacc[i,t-1]*(1-riratewithvacc[i,t])+infectionswithvac[i,t]

twoinfectionwithvacc[i,t]<-twoinfectionwithvacc[i,t-1]+oneinfectionwithvacc[i,t-1]*riratewithvacc[i,t]

firstinfectionwithvacc[i,t]<-oneinfectionwithvacc[i,t]-oneinfectionwithvacc[i,t-1]

}

for(t in 1:nt){

#Number of unique individuals who get infected (i.e. excludes reinfected individuals)

#Without vaccination

#Number of people who get infected

individualsnovac[i,t]<-firstinfectionnovacc[i,t]

symptomaticuniqueIDnovac[i,t]<-symptomaticprob[i]*individualsnovac[i,t]

asymptomaticuniqueIDnovac[i,t]<-(1-symptomaticprob[i])*individualsnovac[i,t]

#Number of treated people and non-treated people

treateduniqueIDnovac[i,t]<-(symptomaticuniqueIDnovac[i,t]*treatSprob[i]+asymptomaticuniqueIDnovac[i,t]*treatASprob[i])*successrate

nontreateduniqueIDnovac[i,t]<-symptomaticuniqueIDnovac[i,t]*(1-treatSprob[i])+asymptomaticuniqueIDnovac[i,t]*(1-treatASprob[i])+(symptomaticuniqueIDnovac[i,t]*treatSprob[i]+asymptomaticuniqueIDnovac[i,t]*treatASprob[i])*(1-successrate)

#With vaccination

#Number of people who don't get infected thanks to vaccination

individualspreventedvaccinated[i,t]<-firstinfectionnovacc[i,t]-firstinfectionwithvacc[i,t]

#Number of people (incl. indirect effect) who don't get infected thanks to vaccination

individualsprevented[i,t]<-individualspreventedvaccinated[i,t]*(1+addtransmittedcase)

symptomaticuniqueID[i,t]<-symptomaticprob[i]*individualsprevented[i,t]

asymptomaticuniqueID[i,t]<-(1-symptomaticprob[i])*individualsprevented[i,t]

treateduniqueID[i,t]<-(symptomaticuniqueID[i,t]*treatSprob[i]+asymptomaticuniqueID[i,t]*treatASprob[i])*successrate

nontreateduniqueID[i,t]<-symptomaticuniqueID[i,t]*(1-treatSprob[i])+asymptomaticuniqueID[i,t]*(1-treatASprob[i])+(symptomaticuniqueID[i,t]*treatSprob[i]+asymptomaticuniqueID[i,t]*treatASprob[i])*(1-successrate)

}

}

#Sequelae (with offset due to delay in developping sequelae) - sequelae applied at the individual level and not at the infection level

for(i in 1:ns){

for(j in 1:nseq){

for(t in 1:sequelaeonset[j]){

sequelae[i,j,t]<-0

sequelaenovac[i,j,t]<-0

}

for(t in 1:nt-sequelaeonset[j]){

sequelae[i,j,t+sequelaeonset[j]]<- sequelaetreatedprob[i,j]*treateduniqueID[i,t]+sequelaenontreatedprob[i,j]*nontreateduniqueID[i,t]

sequelaenovac[i,j,t+sequelaeonset[j]]<- sequelaetreatedprob[i,j]*treateduniqueIDnovac[i,t]+sequelaenontreatedprob[i,j]*nontreateduniqueIDnovac[i,t]

}

}

}

#Cost of the disease:

for(t in 1:nt){

for(i in 1:ns){

#Costs without vaccination

treatmentcostnovac[i,t]<-treatednovac[i,t]*ctreatment[i]/successrate #Division by success rate since treated[i,t] is the number of successfully treated infections

HIVcostnovac[i,t]<-HIVc*HIVindirectnovac[i,t]

productivitycostnovac[i,t]<-treateduniqueIDnovac[i,t]*productivitytreatmentc[i]+nontreateduniqueIDnovac[i,t]*productivityc[i]

HIVproductivitynovac[i,t]<-productivityHIV*HIVindirectnovac[i,t]*(1-percHIVviralsuppress)

#Cost savings from vaccination

treatmentcost[i,t]<-treated[i,t]*ctreatment[i]/successrate

HIVcost[i,t]<-HIVc*HIVindirect[i,t]

HIVproductivity[i,t]<-productivityHIV*HIVindirect[i,t]*(1-percHIVviralsuppress)

productivitycost[i,t]<-nontreateduniqueID[i,t]*productivityc[i]+treateduniqueID[i,t]*productivitytreatmentc[i]

for(j in 1:nseq){

sequelaecosts[i,j,t]<-sequelae[i,j,t]*sequelaec[j]

sequelaecostsnovac[i,j,t]<-sequelaenovac[i,j,t]*sequelaec[j]

}

}

#Discounted costs

#Cost without vaccination

coststreatmentdiscnovac[t]<-sum(treatmentcostnovac[,t])*1/pow((1+rc), t-1)

costsHIVdiscnovac[t]<-sum(HIVcostnovac[,t])*1/pow((1+rc), t-1)

productivityHIVdiscnovac[t]<-sum(HIVproductivitynovac[,t])*1/pow((1+rc), t-1)

costssequelaediscnovac[t]<-sum(sequelaecostsnovac[,,t])*1/pow((1+rc), t-1)

productivitycostdiscnovac[t]<-sum(productivitycostnovac[,t])*1/pow((1+rc), t-1)

#Savings from vaccination

coststreatmentdisc[t]<-sum(treatmentcost[,t])*1/pow((1+rc), t-1)

costsHIVdisc[t]<-sum(HIVcost[,t])*1/pow((1+rc), t-1)

productivityHIVdisc[t]<-sum(HIVproductivity[,t])*1/pow((1+rc), t-1)

costssequelaedisc[t]<-sum(sequelaecosts[,,t])*1/pow((1+rc), t-1)

productivitycostdisc[t]<-(sum(productivitycost[,t])+sum(HIVproductivity[,t]))*1/pow((1+rc), t-1)

costsHCdisc[t]<-coststreatmentdisc[t]+costsHIVdisc[t]+costssequelaedisc[t]

costsSocietaldisc[t]<-costsHCdisc[t]+productivitycostdisc[t]

}

#Utilities lost to the disease (symptoms and sequelae)

for(j in 1:nseq){

sequelaedis[j]<-sequelaedisyr[j]*1/rb*(1+rb-1/pow((1+rb), sequelaeduration[j]-1))

}

for(t in 1:nt){

for(i in 1:ns){

HIVdisutilities[i,t]<-HIVdis*HIVindirect[i,t]

HIVdisutilitiesnovac[i,t]<-HIVdis*HIVindirectnovac[i,t]

for(j in 1:nseq){

#No vaccination

sequelaedisutilitiesnovac[i,j,t]<-sequelaenovac[i,j,t]*sequelaedis[j]*sequelaeimpactprob[j]

symptomsdisutilitiesnovac[i,j,t]<-symptomaticnovac[i,t]*symptomprob[i,j]*symptomsdis[j]

#Prevented from vaccination

sequelaedisutilities[i,j,t]<-sequelae[i,j,t]*sequelaedis[j]*sequelaeimpactprob[j]

symptomsdisutilities[i,j,t]<-symptomatic[i,t]*symptomprob[i,j]*symptomsdis[j]

}

}

disutilities[t]<-sum(symptomsdisutilities[,,t])+sum(sequelaedisutilities[,,t])+sum(HIVdisutilities[,t])

disutilitiesnovac[t]<-sum(symptomsdisutilitiesnovac[,,t])+sum(sequelaedisutilitiesnovac[,,t])+sum(HIVdisutilitiesnovac[,t])

disutilitiesdisc[t]<-disutilities[t]*1/pow((1+rb), t-1)

infectionspreventeddisc[t]<-sum(infectionsprevented[,t])*1/pow((1+rb), t-1)

}

test<-sum(sequelaedisutilities[,,])

#*******************Results summary *************************

#Number of infections

totalinfectionsnovac<-sum(infectionsnovac[,])

totalinfectionsmennovac<-sum(infectionsnovac[1,])

totalinfectionswomennovac<-sum(infectionsnovac[2,])

totalinfectionsprevented<-sum(infectionsprevented[,])

totalinfectionspreventedindirect<-totalinfectionsprevented*addtransmittedcase/(1+addtransmittedcase)

totalinfectionswithvac<-totalinfectionsnovac-totalinfectionsprevented

totalinfectionspreventeddisc<-sum(infectionspreventeddisc[])

#Number of uniquely infected people

percFirstInfections<-sum(firstinfectionnovacc[,])/sum(infectionsnovac[,])

totalindividualsnovac<-sum(individualsnovac[,])

totalindividualspreventedvaccinated <-sum(individualspreventedvaccinated[,])

#Number of sequelae

for(i in 1:ns){

for(j in 1:nseq){

totalsequelae[i,j]<-sum(sequelae[i,j,])

totalsequelaenovac[i,j]<-sum(sequelaenovac[i,j,])

}

}

#Number of HIV cases

totalHIVcasesnovac<-sum(HIVindirectnovac[,])

totalHIVprevented<-sum(HIVindirect[,])

#Disutilites

#No vaccination

totaldisutilitiesnovac<-sum(disutilitiesnovac[])

totalsequelaedisutilitiesnovac<-sum(sequelaedisutilitiesnovac[,,])

totalHIVdisutilitiesnovac<-sum(HIVdisutilitiesnovac[,])

totalPIDdisutilitiesnovac<-sum(sequelaedisutilitiesnovac[2,1,]) #PID

totaleCPPdisutilitiesnovac<-sum(sequelaedisutilitiesnovac[2,3,]) #CPP

totalinfertilitydisutilitiesnovac<-sum(sequelaedisutilitiesnovac[2,4,]) #Infertility

#Disutilities avoided from vaccination

totaldisutilities<-sum(disutilities[])

totaldisutilitiesdisc<-sum(disutilitiesdisc[])

#Costs

#No vaccination

totalcoststreatmentdiscnovac<-sum(coststreatmentdiscnovac[])

totalcostsHIVdiscnovac<-sum(costsHIVdiscnovac[])

totalproductivityHIVdiscnovac<-sum(productivityHIVdiscnovac[])

totalcostssequelaediscnovac<-sum(costssequelaediscnovac[])

totalmedcostsnovac<-totalcoststreatmentdiscnovac+totalcostsHIVdiscnovac+totalcostssequelaediscnovac

totalproductivitycostdiscnovac<-sum(productivitycostdiscnovac[])

totalproductivitycostHIVdiscnovac<-sum(productivityHIVdiscnovac[])

#Cost savings from vaccination

totalcostsHCdisc<-sum(costsHCdisc[])

totalproductivitycostdisc<-sum(productivitycostdisc[])

totalcostsSocietaldisc<-sum(costsSocietaldisc[])

#Justifiable price

totalvalue<-(WTP*totaldisutilitiesdisc+totalcostsSocietaldisc)

justifiableprice<-(WTP*totaldisutilitiesdisc+totalcostsHCdisc)/(sum(cohortvac[])*vaccinationrate*nbdoses)

justifiablepricewithproductivity<-totalvalue/(sum(cohortvac[])*vaccinationrate*nbdoses)

#Key metrics

vaccinationcosts<-sum(cohortvac[])*vaccinationrate*nbdoses*justifiablepricewithproductivity

vaccinationcostsnet<-vaccinationcosts-totalcostsHCdisc

costperinfectionaverted<-vaccinationcostsnet/totalinfectionspreventeddisc

#Justifiable price per category

justifiablepricetreatmentcosts<-sum(coststreatmentdisc[])/(sum(cohortvac[])*vaccinationrate*nbdoses)

justifiablepricesequelaecosts<-sum(costssequelaedisc[])/(sum(cohortvac[])*vaccinationrate*nbdoses)

justifiablepriceHIVcosts<-sum(costsHIVdisc[])/(sum(cohortvac[])*vaccinationrate*nbdoses)

justifiablepricesequelaedisutility<-sum(disutilitiesdisc[])*WTP/(sum(cohortvac[])*vaccinationrate*nbdoses)

}

**#DATA (i.e. input parameters)**

list(nt=60, #number of period under considerations (60 years)

efficmeanduration= 10, #mean duration ofefficacy of the vaccine

efficacyrate= 0.2, # efficacy of the vaccine

vaccinationrate= 0.705, # vaccination rate

addtransmittedcase= 0.5, # the prevention of each case in GC prevents 0.5 case in the general population by interrupting the transmission of gonorrhea

cohortvac= c(2047,1957),

incidence= c(466,354),

nbincgrp=10, # number of age group for which we have incidence

nbcohortspergroup=5 # number of cohorts in each age groups (life data, incidence

#% of cases per age group. Col1:15-19, col2:20-24, col3:25-29, col4:30-34, col5:35-39, col6:40-44 (1=men, 2=women)

#Sum of pctcasesperagegroup>100% since we are interested in 15-64 and not only 15-39

pctcasesperagegroup= structure(.Data= c(0.214,0.379,0.216,0.122,0.068,0.053, 0.031, 0.031, 0.009, 0.009, 0.368,0.380,0.153,0.068,0.031,0.017, 0.007, 0.007, 0.001, 0.001), .Dim=c(2, 10)),

#cohort size per age group. Col1:15-19, col2:20-24, col3:25-29, col4:30-34, col5:35-39, col6:40-44 col7:45-49 col8:50-54 col9:55-59 col10:60-65

cohortsizeagegroup= structure(.Data= c(10691,10960,10917,10067,9542,10172, 10800, 10695,9499, 8447, 10196,10565,10464,10135,9713,10415,11189,11270,10055,8983), .Dim=c(2, 10)),

#death rate Col1:15-19, col2:20-24, col3:25-29, col4:30-34, col5:35-39, col6:40-44, col7:45-49, col8:50-54, col9:55-59, col10: 60-64. col11:65-69, col12:70-74

#http://www.cdc.gov/nchs/data/dvs/deaths_2010_release.pdf

deathrate=structure(.Data= c(69.6E-5, 126.4E-5, 135.7E-5, 147.7E-5, 175.4E-5, 248.4E-5, 401.0E-5, 613.5E-5, 911.2E-5, 1269.2E-5, 1871.3E-5, 2831.9E-5, 28.1E-5, 44.8E-5, 55.7E-5, 72.6E-5, 102.6E-5, 154.3E-5, 248.9E-5, 374.5E-5, 524.5E-5, 781.7E-5, 1222.0E-5,1926.9E-5), .Dim=c(2, 12)),

ns=2, #number of subgroup

symptomaticprob= c(0.5,0.25), #probability of being symptomatic (1=men, 2=women)

treatSprob= c(0.89,0.89), #probability of receiving treatment if symptomatic (1=men, 2=women)

treatASprob= c(0.09,0.4), #probability of receiving treatment if asymptomatic (1=men, 2=women)

successrate=0.97 #probability of that the antibiotic treatment works

HIVprob= 0.00066, # probability that a GC case will facilitate a new case of HIV transmission

percHIVviralsuppress= 0.25, # percentage of HIV patients that achieves viral load suppression

nseq=5, #number of sequelae type and symptoms

# Probability of having sequelae: col1: PID, col2: ectopic pregrancy; col3: chronic pelvic pain; col4:Infertility; col5: Epididymitis

sequelaenontreatedprob= structure(.Data= c(0,0,0,0,0.02, 0.15,0.01125,0.02700,0.03075,0), .Dim=c(2, 5)),

sequelaetreatedprob= structure(.Data= c(0,0,0,0,0, 0.05,0.00375,0.009,0.01025,0), .Dim=c(2, 5)),

# Percentage of woment who want to conceive

sequelaeimpactprob=c(1,1,1,0.772,1),

# Onset of sequelae

sequelaeonset= c(0,5,5,5,0),

ctreatment=c(176.9,169.9), #treatment costs (diagnostics, outpatient, drugs) (1=men, 2=women)

HIVc=325500,# lifetime cost per case of HIV

productivityc= c(39,195), # lost due to sequelae

productivitytreatmentc= c(201.1,201.1), # lost due to sequelae

productivityHIV= 947209, # productitivity lost for each HIV case

sequelaec= c(3420,0,0,0,334), # Costs of sequelae (note: cost of ectopic pregrancy and mortality already in PID

# Probability of having symptoms # colum 1: urethritis:

symptomprob=structure(.Data= c(0.84,0,0,0,0, 0,0,0,0,0), .Dim=c(2, 5)),

symptomsdis= c(0.00285,0,0,0,0), # Disutility of symptoms - colum 1: urethritis

sequelaedisyr= c(0.00837,0.02973,0.083,0.1656,0.00920), # Disutility of sequelae/period col1: PID, col2: ectopic pregrancy; col3: chronic pelvic pain; col4:Infertility; col5: Epididymitis

sequelaeduration= c(1,1,10,10,1), # Disutility of sequelae

HIVdis=6.95, # lifetime number of QALYs lost per case per HIV case

rc=0.03, #cost discount

rb=0.03, #benefit discount

WTP=75000, #willingness to pay per QALY gained

nbdoses=2, #number of doses required to complete the vaccination

reinfectionrate=0.045, #rate of reinfection

)

**#RESULTS**
